# ATACgraph: Profiling Genome-Wide Chromatin Accessibility From ATAC-seq

**DOI:** 10.3389/fgene.2020.618478

**Published:** 2021-01-13

**Authors:** Rita Jui-Hsien Lu, Yen-Ting Liu, Chih Wei Huang, Ming-Ren Yen, Chung-Yen Lin, Pao-Yang Chen

**Affiliations:** ^1^Institute of Plant and Microbial Biology, Academia Sinica, Taipei, Taiwan; ^2^Department of Medicine, Washington University in St. Louis, St. Louis, MO, United States; ^3^Institute of Information Science, Academia Sinica, Taipei, Taiwan

**Keywords:** chromatin accessibility, ATAC-seq, bioinformatics, next-generation sequencing, epigenomics, genomics

## Abstract

Assay for transposase-accessible chromatin using sequencing data (ATAC-seq) is an efficient and precise method for revealing chromatin accessibility across the genome. Most of the current ATAC-seq tools follow chromatin immunoprecipitation sequencing (ChIP-seq) strategies that do not consider ATAC-seq-specific properties. To incorporate specific ATAC-seq quality control and the underlying biology of chromatin accessibility, we developed a bioinformatics software named ATACgraph for analyzing and visualizing ATAC-seq data. ATACgraph profiles accessible chromatin regions and provides ATAC-seq-specific information including definitions of nucleosome-free regions (NFRs) and nucleosome-occupied regions. ATACgraph also allows identification of differentially accessible regions between two ATAC-seq datasets. ATACgraph incorporates the docker image with the Galaxy platform to provide an intuitive user experience via the graphical interface. Without tedious installation processes on a local machine or cloud, users can analyze data through activated websites using pre-designed workflows or customized pipelines composed of ATACgraph modules. Overall, ATACgraph is an effective tool designed for ATAC-seq for biologists with minimal bioinformatics knowledge to analyze chromatin accessibility. ATACgraph can be run on any ATAC-seq data with no limit to specific genomes. As validation, we demonstrated ATACgraph on human genome to showcase its functions for ATAC-seq interpretation. This software is publicly accessible and can be downloaded at https://github.com/RitataLU/ATACgraph.

## Introduction

Chromatin is composed of nucleosomes, which each consisting of a histone octamer core wrapped by 147 bp of DNA (Tsompana and Buck, 2014), and the accessibility of chromatin can be assessed based on the nucleosome density; dense nucleosome regions (closed regions) are tightly packed, whereas loose nucleosome regions (open regions) are more accessible. The chromatin accessibility is directly associated with gene expression and biological functions such as cellular differentiation and reprogramming (Jung et al., 2017). In plants, chromatin accessibility is often associated with intron retention (Reddy et al., 2012; Oka et al., 2017; Ullah et al., 2018), which plays an important role in the generation of gene product diversity.

Several sequencing strategies such as MNase-seq (Rizzo and Sinha, 2014), DNase-seq (Song and Crawford, 2010), FAIRE-seq (Giresi et al., 2007), and ATAC-seq (Buenrostro et al., 2013) have been developed for the assessment of chromatin structure. MNase-seq is an endo-exonuclease that digests linker DNA between nucleosomes and unprotected DNA to reveal the position of nucleosomes. MNase-seq selects DNA sequences wrapping around the core histones (Barski et al., 2007); hence, a high density of MNase-seq reads represents a closed chromatin. DNase-seq digests with DNase I endonuclease (Buenrostro et al., 2013), and the resulting DNA fragments correspond to open chromatin regions. FAIRE-seq uses formaldehyde and phenol–chloroform extraction separation to obtain exposed DNA and to reveal regions of open chromatin. While MNase-seq identifies nucleosome-dense regions, DNase-seq and FAIRE-seq are used to identify open chromatin regions. However, these techniques have hurdles that are requiring high sample quantity and processing time. MNase-seq requires 10^7^ to 10^8^ cells, and the micrococcal nuclease has sequence-specific biases such as AT-rich. DNase-seq also requires 10^6^ of cells, and the endonuclease used in DNase-seq also has sequence biases. With a similar requirement of a sample amount of millions of cells, the resolution of FAIRE-seq is lower than that of other techniques (Hsu et al., 2018). Recently, a technique named Assay for transposase-accessible chromatin with sequencing (ATAC-seq) was developed to identify the chromatin accessibility regions to complement other next-generation sequencing (NGS)-based techniques (Buenrostro et al., 2013). ATAC-seq utilizes Tn5 transposase to cleave DNA fragments in open chromatin regions and simultaneously integrates adaptor sequences into these regions for the detection of open chromatin structures (Buenrostro et al., 2013). ATAC-seq requires less material (500–50,000 cells) of tissue/cells and sample-processing time, as opposed to the sequencing of MNase-seq, DNase-seq, and FAIRE-seq. ATAC-seq libraries preparation takes only within 1 day, whereas DNase-seq, MNase-seq, and FAIRE-seq usually require 3–4 days. ATAC-seq is thus a feasible tool for profiling chromatin accessibility.

To date, for different target audiences, several comprehensive pipelines have been developed for processing ATAC-seq data. GUAVA (Divate and Cheung, 2018) and I-ATAC (Ahmed and Ucar, 2017) with a graphical user interface (GUI) are adapted to bench biologists. NucleoATAC (Schep et al., 2015) is the earliest tool to predict nucleosome positions and occupancy from ATAC-seq data. The ENCODE ATAC-Seq Pipeline (Kundaje, 2018) is a pipeline used for quality assessment of ATAC-seq, producing alignments and measures of enrichment. ATACseqQC (Ou et al., 2018) and esATAC (Wei et al., 2018) are both R/Bioconductor packages covering raw data alignment, performing quality control (QC) functions and transcription factor foot printing. nf-core/atacseq (Ewels et al., 2020) is a pipeline built by Nextflow, a workflow tool, to run multiple tasks across multiple computing infrastructures.

Most of these tools require users to be able to work with the command-line interface (CLI) on Linux-like systems for installation and general functions; and some of the GUI tools are no longer updated/maintained regularly. Here, we developed ATACgraph, a comprehensive software tool designed for integrated analysis of ATAC-seq, covering QC functions and downstream analyses. In addition to the functions from all existing tools, ATACgraph can also detect nucleosome-free regions (NFRs) and nucleosome-occupied regions, and the integrative analysis of ATAC-seq with other NGS data. ATACgraph incorporates the docker image with the Galaxy platform, an open-source platform with access via a web-based interface to provide an intuitive user experience. For the ease of user, ATACgraph provides existing workflows that can be executed on the Galaxy platform. Advanced users can easily create customized pipelines through flexible interfaces in the ATACgraph Galaxy platform.

## Methods

**FIGURE 1:**
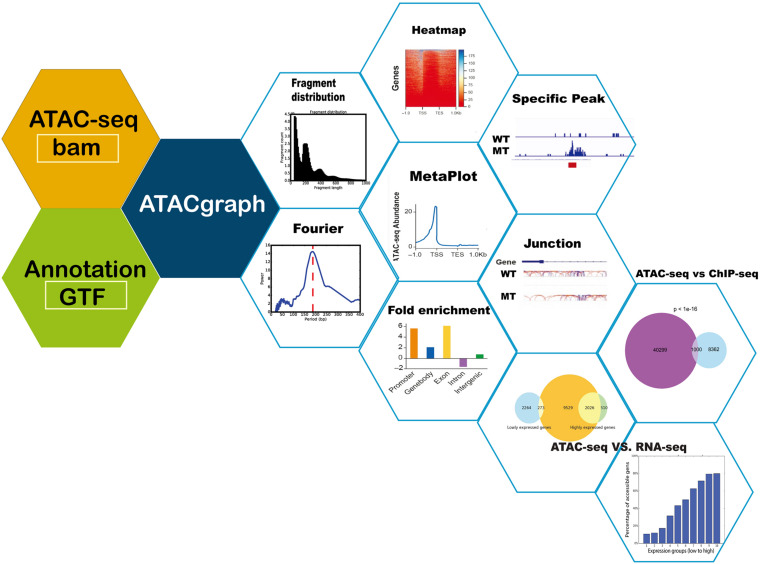
Schematic diagram of ATACgraph. ATACgraph workflow contains 12 modules; most of them are processing independently, and some are sequential processing containing two modules. It can also implement Galaxy-docker to construct pipelines. All modules can handle paired-end and single-end reads; only fragment distribution, FFT (fast Fourier transform), and peak calling need paired-end reads to process. To run all modules of ATACgraph workflow, users provide paired-end aligned BAM file and gene annotation file. The first step is to remove mitochondria or plastid DNA reads and then generate two figures to check quality control (QC) and contain a fragment distribution bar plot and FFT figure to calculate the period of periodicity in the fragment length distribution analysis. Based on fragment distribution figures, the user can set up a cutoff for selecting fragment length for the following analysis. After selecting fragment length, ATACgraph can generate junction tracks, peak calling, visualization for accessible regions, and enrichment analysis for these regions. ATACgraph also include comparisons between two groups of ATAC-seq. Furthermore, ATACgraph also offers a module to compare accessible regions identified using another next-generation sequencing (NGS) method, and output includes a Venn diagram showing the number of overlapping regions and a list of the locations of these regions. ATACgraph generates a bar plot and Venn diagram to show the relationship between accessibility and gene expression level when comparing ATAC-seq and RNA-seq.

ATACgraph is a Python software that performs various ATAC-seq analyses and can be divided into three major components (see Figure 1): (1) preprocessing, which produces intermediate outputs before analysis, such as the reads after filtering out mitochondrial DNA and quality assessment of aligned ATAC-seq datasets; (2) profiling, which is to detect accessible regions (also referred to as peaks) and to visualize the accessibilities; and (3) comparison, which includes discovering differential peaks between two groups of ATAC-seq datasets. Integrating ATAC-seq with other high-throughput sequencing technologies such as chromatin immunoprecipitation sequencing (ChIP-seq) and RNA-seq can mutually validate the reliability of each other within the same experimental system. All modules are implemented in the ATACgraph modules and are listed in Supplementary Table S1.

### Preprocessing of Alignment Reads

Alignments of ATAC-seq in BAM files are first imported into ATACgraph for filtering out mitochondrial DNA. Removing mitochondrial reads is an important step because up to 20–80% of sequences in ATAC-seq arise from the mitochondrial genome and might influence the subsequent analyses. ATACgraph can generate filtered BAM files by removing reads derived from mitochondrial and plastid DNA. Next, ATACgraph is able to plot the fragment size distribution from the filtered BAM file. The module implemented the fast Fourier transform (FFT) algorithm to estimate the period of the fragment length distribution.

### Identifying Accessible Regions

One of the major steps of ATACgraph data analysis is to identify accessible regions and is the basis for subsequent analyses. ATACgraph incorporates MACS2 (Feng et al., 2012) to identify accessible regions, also known as the peak locations. Two modes, peak calling from full-extended fragments and peak calling from integration sites, are applied for identifying the chromatin accessibility in this module. These two modes are used according to different regions; peak calling from full-extended fragments identifies nucleosome-occupied regions, whereas the peak calling from integration sites defines the accessible regions (nucleosome free). The details are listed in Supplementary Table S2. The peak calling step also allows ATAC-seq control bam file. To study the enrichment of these accessible regions in specific genomic features such as promoter, gene body, exon, intron, 5′UTR, CDS, 3′UTR, and intergenic region (IGR), ATACgraph computes the enrichment of ATAC-seq peaks at these genomic features against the genome background and generates a bar chart to indicate the enrichment status using Equation 1.

(1) $$l\,o\,g_{2}\,\left(\frac{\frac{S_{P\,f}}{S_{P}}}{\frac{S_{g\,f}}{S_{g}}}\right)$$

where *S*_*P*_ and *S*_*g*_ represent the size of all peaks and the genome size in base pairs, respectively; *S*_*Pf*_ is the size of overlapping regions between the peaks and the genomic feature; and *S*_*gf*_ is the size of the genomic feature in the genome. A higher fold enrichment value indicates a high tendency to contain open chromatin in the specific genomic feature. To visualize aggregated signals of chromatin accessibility surrounding genes or peaks, ATACgraph generates two kinds of heatmap and metaplot. One is to show the accessibility around genes (downstream and upstream 1 kb of each gene). After peak calling, the abundance of each peak will be normalized into reads per kilobase per million mapped reads (RPKM). The heatmap will be generated based on these RPKM values, which shows the ATAC-seq abundance (accessibility) around genes. The metaplot proceeds similarly as a condensed version of the heatmap. Another one is to show the accessibility around peaks. The heatmap and metaplot are processed and normalized as described above (same as genes). To improve data visualization in order to recognize NFRs, ATACgraph allows simplified visualization of read distribution along with genomic regions of interest after loading into Integrative Genomics Viewer (IGV) (Robinson et al., 2017). ATACgraph generates a fragment junction track from ATAC-seq reads to be browsed on IGV; a junction connects the start and end positions of a paired-end read in this track. Since the fragment size distribution of ATAC-seq has a clear periodicity at approximately 200 bp, indicating that long fragments (>200 bp) are protected by integer multiples of nucleosomes (Buenrostro et al., 2013), the fragment lengths below 150 bp were marked as blue junctions and fragments lengths above 150 bp were marked as red junctions—which represent possibly NFRs and nucleosome-occupied regions, respectively.

### Identifying Differential Accessibilities

To identify differentially accessible regions between samples, ATACgraph has two analyses to detect differentially accessible regions, based on the nature of the genomic regions: one is the differentiated peaks (peaks with a strong difference in read abundance) between two groups of ATAC-seqs; another one is differentially accessible promoters. To identify differentiated peaks between two groups, ATACgraph provides two methods. If replicates are available, ATACgraph utilizes an abundance-based approach to identify the differentially accessible regions between two groups of samples. ATACgraph embodies the BEDTools multiinter module (Quinlan and Hall, 2010) to identify peaks candidates, and at these candidate locations, ATACgraph calculates ATAC-seq abundance from bigWig files. Differentially accessible peaks were defined by default settings as the peak abundance difference is ≥2-fold, and the *p*-value of the *t*-test is <0.05. For those data with no replicate, ATACgraph is able to incorporate Irreproducible Discovery Rate (IDR) (Li et al., 2011), a published method to measure the reproducibility of findings identified from replicate experiments, for specific peak identification. The files of high confidence peak locations were generated between biological replicates within one group using IDR. By comparing two confidence peak files, the specific peaks were defined as those where the location of each peak is unique in each group of ATAC-seq. To identify differentially accessible promoters, ATACgraph incorporates the Gaussian Mixture Model (GaussianMixture module from the scikit-learn package in Python, or GMM) in the bi-variate correlation plot (Pedregosa et al., 2011) to examine the abundance of promoter regions to find out the outliers that are considered to differentiate promoters (i.e., differentially accessible promoters). As the default setting, differentially accessible promoters are defined as *p*-value < 0.05 in GMM. ATACgraph is also capable of comparing peaks (open regions) from ATAC-seq with those from other high-throughput sequencing technologies such as ChIP-seq, MNase-seq, and DNase-seq. ATACgraph is able to plot the overlap between two sets of peaks from ATAC-seq and another sequencing dataset to provide the *p*-value of the number of overlapping peaks with a hypergeometric test. Also, this module will provide a BED file that indicates the locations of overlapping peaks and generate a Venn diagram showing the number of overlapping regions and *p*-value. Lastly, a list of genes and promoters associated with these overlapping peaks will be generated. To better reveal the association between gene expression and chromatin accessibility, ATACgraph is able to analyze the tendency of accessible (opened) genes through RNA-seq data. ATACgraph is able to separate genes into groups according to their gene expression levels (i.e., RPKM) and generates a bar plot to show the percentage of accessible genes in each expression group. Furthermore, ATACgraph is able to plot the overlapping genes between accessible genes and lowly/highly expressed genes with a Venn diagram.

## Results

ATACgraph is available through both the browser-based interface and the standalone version for command-line usage. On the browser-based interface, an aligned ATAC-seq BAM file and gene annotation file are uploaded on the Galaxy platform as corresponding inputs. The Galaxy installation tutorial can be accessed via https://hub.docker.com/r/lsbnb/galaxy_atacgraph. To simplify the scenarios of various analyses that users may execute, we have constructed several built-in workflows into the Galaxy platform (Supplementary Figure S1). In the standalone version, ATACgraph can be executed in the local Unix/Linux environment, and the tutorial is provided at the GitHub repository^1^.

### Demonstration of ATACgraph With Human Data

**FIGURE 2:**
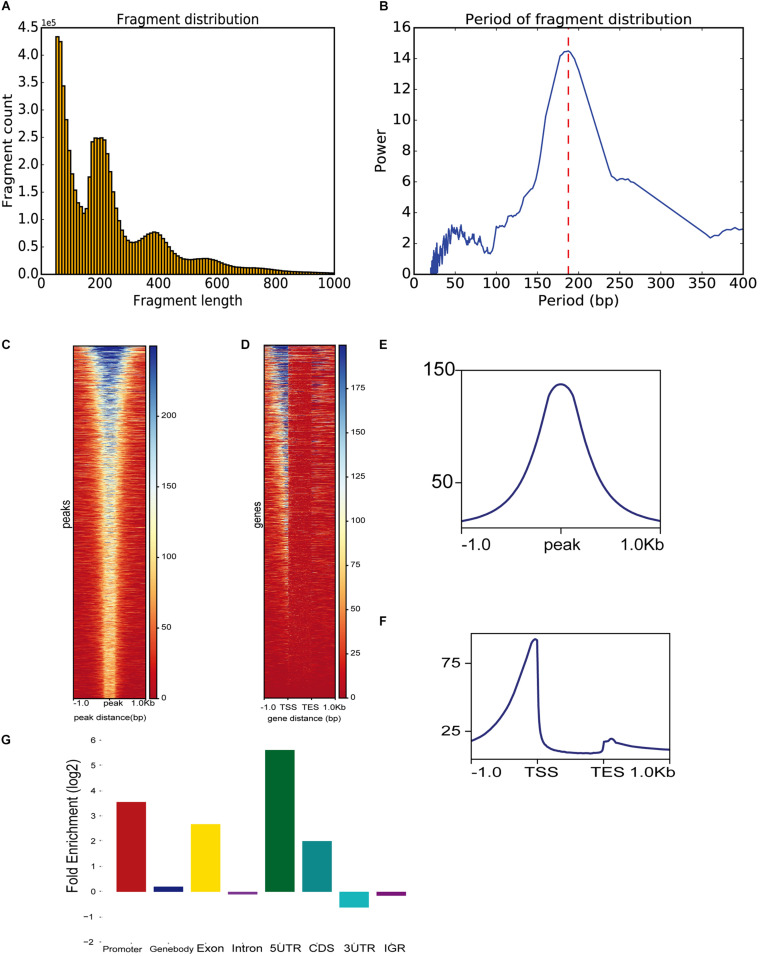
ATAC-seq data-specific analyses generated by ATACgraph. **(A)** ATAC-seq fragment length distribution of human embryonic stem cell (hESC) samples. Histogram shows periodicity signal of nucleosomes. **(B)** The period of fragment length distribution using fast Fourier transform (FFT) algorithm. Heatmaps show accessibility around ATAC-seq peaks **(C)** and whole genes **(D)** of trim28 mutant ATAC-seq reads in profile map. Metaplots show the abundance around ATAC-seq peaks **(E)** or genes **(F)**. **(G)** Fold enrichment of trim28 mutant accessible regions on genomic regions (promoter, gene-body, exon, intron, 5′UTR, CDS, 3′UTR, and IGR). TSS, transcription start site; TES, transcriptome end sites.

To demonstrate ATACgraph on real data, we downloaded ATAC-seq data of wild-type (WT) and *trim28*-mutant human embryonic stem cells (hESCs) (Tao et al., 2018) (GSE99215), both of which have two biological replicates. Raw reads of ATAC-seq were mapped to human hg19 reference genome with Bowtie2 (Langmead and Salzberg, 2012), and the mapping commands are shown in Supplementary Table S3. Furthermore, the output BAM files were loaded into ATACgraph for processing. Duplicated reads and reads aligned to mitochondrial/plastid DNA are removed. ATACgraph generates a figure of the DNA fragment length distribution and computed the periodicity in the fragment length distribution with the FFT algorithm. The distribution figure of hESC ATAC-seq data exhibits a sharp peak at <150 bp (NFR) and three peaks at >150 bp that represent mono-nucleosomes, di-nucleosomes, and tri-nucleosomes (Figure 2A). The FFT analysis computed that the period of the fragment length distribution was approximately 190 bp (Figure 2B). To evaluate the quality of the ATAC-seq peaks, ATACgraph generated a heatmap and a metaplot for representing the ATAC-seq abundance near the peak regions (Figure 2C), which was enriched at the center of the predicted peak locations. The metaplot of the peaks (Figure 2E) also displayed a clear signal enriched at the center of the peak locations. The same analysis was applied to investigate the chromatin accessibility around coding genes. ATACgraph analyzes the ATAC-seq abundance in the gene body as well as the flanking regions. The accessibility of a whole gene set is displayed as a heatmap in which the rows are the genes ranked from high to low chromatin accessibility and the colors are coded based on ATAC-seq abundance (Figure 2D). The accessible regions are located before the transcription start sites (TSSs) in two-thirds of the genes, and no accessible regions are found within the gene bodies. Furthermore, the ATAC-seq abundance is clearly enriched at promoters close to TSSs, depleted in the gene body, and slightly enriched after the transcription end sites (TESs) (Figure 2F).

To detect the genomic distribution of open regions, ATACgraph generates a fold-enrichment graph of the enriched peaks at eight genomic features. The enrichment of the identified accessible regions at these features was determined as the frequency relative to that found on a random distribution of the same features across the genome (see section “Methods”). The hESC promoters were enriched with accessible regions, likely because promoters provide binding sites for transcription factors, activators, and other DNA-binding proteins. Overall, 5′UTRs are accessible, whereas introns are not (Figure 2G), consistent with Wu et al. (2018).

**FIGURE 3:**
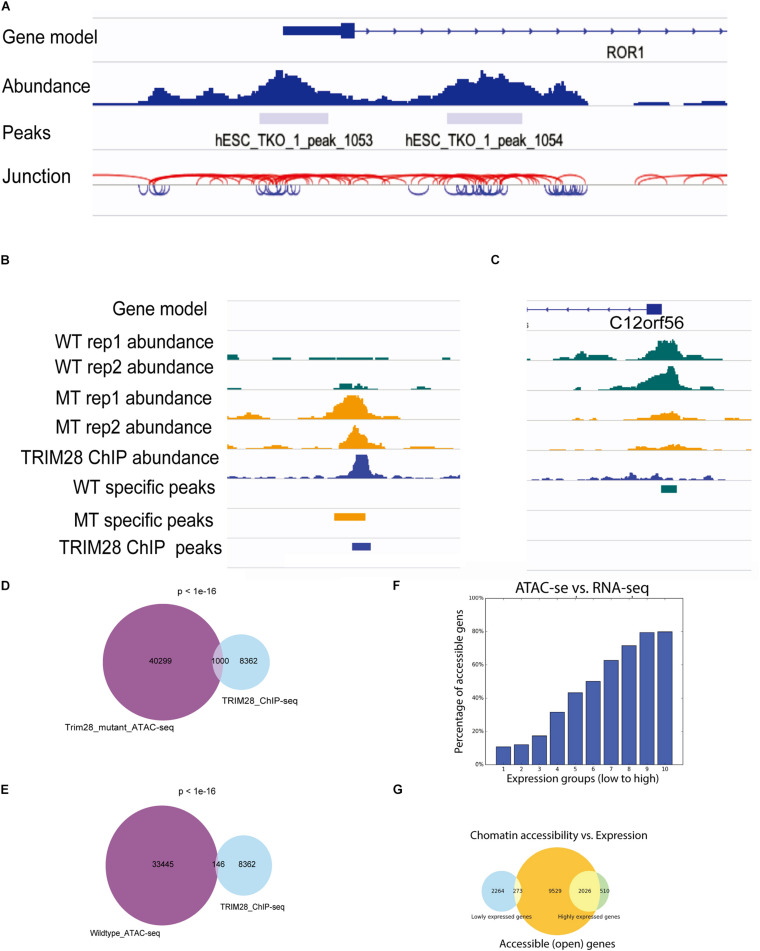
Visualization of ATAC-seq tracks for the identification of accessible chromatin regions. **(A)** ATAC-seq abundance and peaks revealed the positions of accessible chromatin regions around a gene. ATAC fragment junction track shows long (>150 bp) and short (<150 bp) fragments with red and blue lines, respectively. The regions of blue lines suggest nucleosome-free regions, whereas the regions of red lines indicate nucleosome-occupied regions. **(B,C)** Human ATAC-seq data in wild-type (WT) and *TRIM28* mutant (MT). TRIM28 Chip-seq track indicates the location of TRIM28 binding. The abundance tracks show differential abundance between MT and WT tracks at the binding site. **(B)** The region with more accessible in MT. **(C)** The region with less accessible in MT. **(D)** Venn diagram shows 1,000 peaks of overlap between *TRIM28* mutant ATAC-seq peaks and TRIM28 ChIP-seq peaks. **(E)** Venn diagram only shows 146 overlapping peaks between wild-type ATAC-seq without TRIM28 ChIP-seq peaks. **(F)** The bar plot shows that highly expressed genes tend to have a higher percentage of accessible genes than lowly expressed genes. **(G)** The Venn diagram shows the overlapping genes between accessible regions and highly/lowly expression genes.

To facilitate the visualization of ATAC-seq data in IGV, ATACgraph provides junction track files in standard BED. In the junction track, a junction connects the start and end positions of a paired-end read. The short (<150 bp) and long (>150 bp) reads inferred from the fragments of accessible regions between nucleosomes (i.e., NFR); and those across one or more nucleosomes are displayed in blue and red colors, respectively. The junction track for the human data shows that regions with dense blue junctions represent NFRs and are often peak regions (Figure 3A).

There were 33,445 and 43,721 peaks detected from the WTs and 40,299 and 37,828 peaks were detected in *trim28* samples. ATACgraph identified 1,565 peaks that showed significantly increased accessibility and 3,246 peaks showing significantly decreased accessibility in the *trim28* mutant (Figure 3B, MT-specific peaks). A previous study showed that binding to TRIM28 decreases the chromatin accessibility of TE regions (Tao et al., 2018). To explore the association between protein binding and chromatin accessibility, we compared peaks between the ATAC-seq and the TRIM28 (KAP1) ChIP-seq (accession number GSE75868). In Figure 3B, the binding positions for TRIM28 are marked as a peak (TRIM28 ChIP peaks), and the *trim28* mutant exhibited a peak at these binding regions (MT-specific peaks), indicating that the binding to TRIM28 increases the accessibility of the *trim28* mutant—that is to say, binding to TRIM28 decreases chromatin accessibility (Tao et al., 2018). In addition, a region where the WT abundance of ATAC-seq was higher than that of the *trim28* mutant was identified to have exhibited no binding to TRIM28 (Figure 3C). Therefore, we expected to observe many overlapping peaks between the TRIM28 ChIP*-*seq and *trim28* ATAC-seq data and less overlap between the TRIM28 ChIP*-*seq and WT ATAC-seq. The *trim28* mutant ATAC-seq sample revealed 1,000 overlapping peaks between the *trim28* ATAC-seq and TRIM28 ChIP-seq (Figure 3D), whereas only 146 overlapping peaks were found between the WT ATAC-seq and TRIM28 ChIP*-*seq (Figure 3E). The chromatin accessibility is directly associated with gene expression and biological functions such as cellular differentiation and reprogramming (Jung et al., 2017). To explore the relationship between chromatin accessibility and gene expression, we compared the ATAC-seq with RNA-seq in human hESC (accession number GSE75868). The highly expressed genes are expected to contain more accessible genes than the lowly expressed genes. In Figure 3F, genes are equally grouped according to their gene expression levels (i.e., RPKM). Highly expressed genes tend to have a higher percentage of accessible genes than lowly expressed genes. In Figure 3G, the Venn diagram shows that 2,026 out of 2,536 highly expressed genes contain accessible regions, whereas only 273 out of 2,537 lowly expressed genes contain accessible regions. These analyses successfully demonstrate the function of TRIM28 and indicate the high quality of these ATAC-seq libraries.

### Features Comparison With Other Bioinformatics Software

We compared the functional features of ATACgraph with seven popular tools of ATAC-seq data analysis. As summarized in Table 1, the comparison is summarized into categories of user interface, data processing and QC, peak analysis, and the integrative analysis and visualization. Among the eight popular tools that are evaluated, ATACgraph and GUAVA (Divate and Cheung, 2018) cover most features, and the other six tools only have very few functions. In the category of user interface, ATACgraph provides both browser-based interface (Galaxy-docker) and CLI, while GUAVA only provides GUI that does not allow for batch processing. To assist data QC, ATACgraph provides FFT analysis to calculate the periodicity of fragment length that can be very useful to show if the ATAC-seq data clearly reveal the nucleosome turns, hence, the chromatin structures, and this similar feature is not available in GUAVA. In the visualization and integrative analysis, ATACgraph provides several unique features including visualization of accessibility around genes and peaks and compares ATAC-seq peaks with other NGS data that are not seen in GUAVA. In brief, compared with current other tools, ATACgraph provides comprehensive functionalities unique for ATAC-seq analysis.

**TABLE 1:** Features summary of ATAC-seq bioinformatics tools.

**Categories**	**Features**	**ATACgraph**	**NucleoATAC (2015)**	**I-ATAC (2017)**	**GUAVA (2018)**	**ATACseqQC (2018)**	**ENCODE ATAC-Seq Pipeline (2018)**	**esATAC (2018)**	**nf-core/atacseq (2020)**
**User interface**	Interface	Galaxy-docker/CLI	CLI	GUI	GUI	CLI	CLI	CLI	Docker/CLI
**Data preprocessing**	Alignment	–	–	Yes	Yes	–	Yes	Yes	Yes
**and quality check**	Removing mitochondria DNA	Yes	–	–	Yes	Yes	Yes	Yes	Yes
	Fragment size selection	Yes	–	–	Yes	–	–	–	–
	Fragment length distribution plot	Yes	–	–	Yes	–	–	Yes	
	Fast Fourier transform (FFT) analysis	Yes	–	–	–	–	–	–	–
**Peak analysis**	Peak calling	Yes	–	Yes	Yes	–	–	Yes	Yes
	Identifying differentially enriched peaks	Yes	–	–	Yes	–	–	Yes	Yes
**Visualization and**	Enrichment in genomic feature	Yes	Yes	Yes	Yes	Yes	–	Yes	Yes
**integrative analysis**	Visualization of accessibility around genes and peaks	Yes	–	Yes	–	–	Yes	–	Yes
	Visualization of ATAC-seq data tracks	Yes	–	–	Yes	Yes	–	–	–
	Comparing ATAC-seq peaks with other NGS	Yes	–	–	–	–	–	–	–
	Comparing ATAC-seq peaks containing genes with expression	Yes	–	–	–	–	–	–	–
	Transforming GTF annotation to bed	Yes	–	–	–	–	–	–	–
	Nucleosome positing	–	Yes	–	–	Yes	–	–	–
	Transcription factor foot printing	–	Yes	–	–	Yes	–	Yes	–

*Yes, available; “–”, not available; CLI, command-line interface; GUI, graphical user interface.*

## Discussion

ATACgraph is specifically devised for the analysis of post-alignment ATAC-seq, which generates figures for data QC, global patterns, peak calling, gene-level profiling, and statistical analyses, and creates unique tracks for genome-wide visualization. ATACgraph is capable of profiling ATAC-seq data from an individual sample and comparing the accessibilities between multiple samples. In comparison with other bioinformatics tools, ATACgraph is a standalone program, which incorporates most features specifically for analyzing ATAC-seq data. Furthermore, this program can also determine the occurrence of peaks between ATAC-seq data and other NGS data. Also, ATACgraph is a comprehensive tool that can be performed at the Galaxy platform, which provides existing workflows or customized pipelines that allow users to explore ATAC-seq data efficiently.

## System Requirements

ATACgraph operates as a standalone program on computers with Linux operation system.

## Data Availability Statement

Publicly available datasets were analyzed in this study. This data can be found here: GSE99215.

## Author Contributions

P-YC designed the study. RL, M-RY, and Y-TL designed the software and performed the bioinformatics analyses. CH and C-YL constructed the Galaxy platform. RL, Y-TL, M-RY, and P-YC wrote the manuscript. All the authors read and approved the final manuscript.

## Conflict of Interest

The authors declare that the research was conducted in the absence of any commercial or financial relationships that could be construed as a potential conflict of interest.

## Footnotes

1 https://github.com/RitataLU/ATACgraph/blob/master/Tutorial.md

## Supplementary Material

Supplementary Figure 1 Seven workflows of ATACgraph Galaxy-docker for ATAC-seq paired-end data analysis, **(A)** Filtering reads overlapping with blacklist regions. **(B)** Filtering reads chromosome and generates figures of fragment distribution and Fast Fourier analysis. **(C)** Filtering reads chromosome and creating junction files for IGV visualization. **(D)** Defining the differential peaks with specific fragments size. **(E)** Integrating ATAC-seq with other NGS data. **(F)** Integrating ATAC-seq with RNA-seq. **(G)** Visualizing the chromatin accessibility and the fold enrichment of open regions in genomic features.

Supplementary Table 1 Functions implemented in the ATACgraph.

Supplementary Table 2 Summary of suggested parameters for fragment length selection and peak calling.

Supplementary Table 3 Command list for mapping steps.

## Abbreviations

ATAC-seq: assay for transposase-accessible chromatin using sequencing
ChIP-seq: chromatin immunoprecipitation sequencing
NGS: next-generation sequencing
NFR: nucleosome-free region
MNase-seq: micrococcal nuclease sequencing
DNase-seq: sequencing of DNase I hypersensitive sites
FAIRE-seq: formaldehyde-assisted isolation of regulatory elements
FFT: fast Fourier transform
TSS: transcription start site
TES: transcription end site
5’UTR: 5’ untranslated region
CDS: coding sequence
3’UTR: 3’ untranslated region
GMM: Gaussian Mixture Model
IDR: Irreproducible Discovery Rate
GUI: graphical user interface
CLI: command-line interface.

## References

[B1] Ahmed Z. Ucar D. (2017). I-Atac: interactive pipeline for the management and pre-processing of Atac-seq samples. *PeerJ* 5:e4040. 10.7717/peerj.4040 29181276 PMC5702251

[B2] Barski A. Cuddapah S. Cui K. Roh T. Y. Schones D. E. Wang Z. (2007). High-resolution profiling of histone methylations in the human genome. *Cell* 129 823–837. 10.1016/j.cell.2007.05.009 17512414

[B3] Buenrostro J. D. Giresi P. G. Zaba L. C. Chang H. Y. Greenleaf W. J. (2013). Transposition of native chromatin for fast and sensitive epigenomic profiling of open chromatin, DNA-binding proteins and nucleosome position. *Nat. Methods* 10 1213–1218. 10.1038/nmeth.2688 24097267 PMC3959825

[B4] Divate M. Cheung E. (2018). GUAVA: a graphical user interface for the analysis and visualization of ATAC-seq data. *Front. Genet.* 9:250. 10.3389/fgene.2018.00250 30065749 PMC6056626

[B5] Ewels P. A. Peltzer A. Fillinger S. Patel H. Alneberg J. Wilm A. (2020). The nf-core framework for community-curated bioinformatics pipelines. *Nat. Biotechnol.* 38 276–278. 32055031 10.1038/s41587-020-0439-x

[B6] Feng J. Liu T. Qin B. Zhang Y. Liu X. S. (2012). Identifying ChIP-seq enrichment using MACS. *Nat. Protoc.* 7 1728–1740. 10.1038/nprot.2012.101 22936215 PMC3868217

[B7] Giresi P. G. Kim J. Mcdaniell R. M. Iyer V. R. Lieb J. D. (2007). FAIRE (Formaldehyde-Assisted Isolation of Regulatory Elements) isolates active regulatory elements from human chromatin. *Genome Res.* 17 877–885. 10.1101/gr.5533506 17179217 PMC1891346

[B8] Hsu F. M. Gohain M. Albrecht S. Huang Y. J. Liao J. L. Kuang L. Y. (2018). Dynamics of the methylome and transcriptome during the regeneration of rice. *Epigenomes* 2:14.

[B9] Jung S. Angarica V. E. Andrade-Navarro M. A. Buckley N. J. Del Sol A. (2017). Prediction of chromatin accessibility in gene-regulatory regions from transcriptomics data. *Sci. Rep.* 7:4660. 10.1038/s41598-017-04929-6 28680085 PMC5498635

[B10] Kundaje A. (2018). *ENCODE ATAC-Seq Pipeline*. Available online at: https://github.com/ENCODE-DCC/atac-seq-pipeline (accessed April 7, 2020).

[B11] Langmead B. Salzberg S. L. (2012). Fast gapped-read alignment with Bowtie 2. *Nat. Methods* 9 357–359. 10.1038/nmeth.1923 22388286 PMC3322381

[B12] Li Q. Brown J. B. Huang H. Bickel P. J. (2011). Measuring reproducibility of high-throughput experiments. *Ann. Appl. Statist.* 5 1752–1779.

[B13] Oka R. Zicola J. Weber B. Anderson S. N. Hodgman C. Gent J. I. (2017). Genome-wide mapping of transcriptional enhancer candidates using DNA and chromatin features in maize. *Genome Biol.* 18:137. 10.1186/s13059-017-1273-4 PMC5522596 28732548

[B14] Ou J. Liu H. Yu J. Kelliher M. A. Castilla L. H. Lawson N. D. (2018). ATACseqQC: a Bioconductor package for post-alignment quality assessment of ATAC-seq data. *BMC Genom.* 19:169. 10.1186/s12864-018-4559-3 29490630 PMC5831847

[B15] Pedregosa F. Varoquaux G. Gramfort A. Michel V. Thirion B. Grisel O. (2011). Scikit-learn: machine learning in Python. *J. Mach. Learn. Res.* 12 2825–2830.

[B16] Quinlan A. R. Hall I. M. (2010). BEDTools: a flexible suite of utilities for comparing genomic features. *Bioinformatics* 26 841–842. 10.1093/bioinformatics/btq033 20110278 PMC2832824

[B17] Reddy A. S. N. Rogers M. F. Richardson D. N. Hamilton M. Ben-Hur A. (2012). Deciphering the plant splicing code: experimental and computational approaches for predicting alternative splicing and splicing regulatory elements. *Front. Plant Sci.* 3:18. 10.3389/fpls.2012.00018 22645572 PMC3355732

[B18] Rizzo J. M. Sinha S. (2014). Analyzing the global chromatin structure of keratinocytes by MNase-seq. *Methods Mol. Biol.* 1195 49–59. 10.1007/7651_2014_77 24676786

[B19] Robinson J. T. Thorvaldsdottir H. Wenger A. M. Zehir A. Mesirov J. P. (2017). Variant review with the integrative genomics viewer. *Cancer Res.* 77 e31–e34. 10.1158/0008-5472.CAN-17-0337 29092934 PMC5678989

[B20] Schep A. N. Buenrostro J. D. Denny S. K. Schwartz K. Sherlock G. Greenleaf W. J. (2015). Structured nucleosome fingerprints enable high-resolution mapping of chromatin architecture within regulatory regions. *Genome Res.* 25 1757–1770. 10.1101/gr.192294.115 26314830 PMC4617971

[B21] Song L. Crawford G. E. (2010). DNase-seq: a high-resolution technique for mapping active gene regulatory elements across the genome from mammalian cells. *Cold Spring Harb. Protoc.* 2010:pdb prot5384. 10.1101/pdb.prot5384 20150147 PMC3627383

[B22] Tao Y. Yen M. R. Chitiashvili T. Nakano H. Kim R. Hosohama L. (2018). TRIM28-regulated transposon repression is required for human germline competency and not primed or naive human pluripotency. *Stem Cell Rep.* 10 243–256. 10.1016/j.stemcr.2017.11.020 29290627 PMC5768987

[B23] Tsompana M. Buck M. J. (2014). Chromatin accessibility: a window into the genome. *Epigenet. Chrom.* 7:33. 10.1186/1756-8935-7-33 25473421 PMC4253006

[B24] Ullah F. Hamilton M. Reddy A. S. N. Ben-Hur A. (2018). Exploring the relationship between intron retention and chromatin accessibility in plants. *BMC Genom.* 19:21. 10.1186/s12864-017-4393-z 29304739 PMC5756433

[B25] Wei Z. Zhang W. Fang H. Li Y. D. Wang X. W. (2018). esATAC: an easy-to-use systematic pipeline for ATAC-seq data analysis. *Bioinformatics* 34 2664–2665. 10.1093/bioinformatics/bty141 29522192 PMC6061683

[B26] Wu J. Y. Xu J. W. Liu B. F. Yao G. D. Wang P. Z. Lin Z. L. (2018). Chromatin analysis in human early development reveals epigenetic transition during ZGA. *Nature* 557 256–260. 29720659 10.1038/s41586-018-0080-8

