# Supplementary figures and images for: ATACgraph: Profiling Genome-Wide Chromatin Accessibility From ATAC-seq
Source: Front Genet. 2021 Jan 13;11:618478. doi: 10.3389/fgene.2020.618478 (PMC7874078; doi:10.3389/fgene.2020.618478)

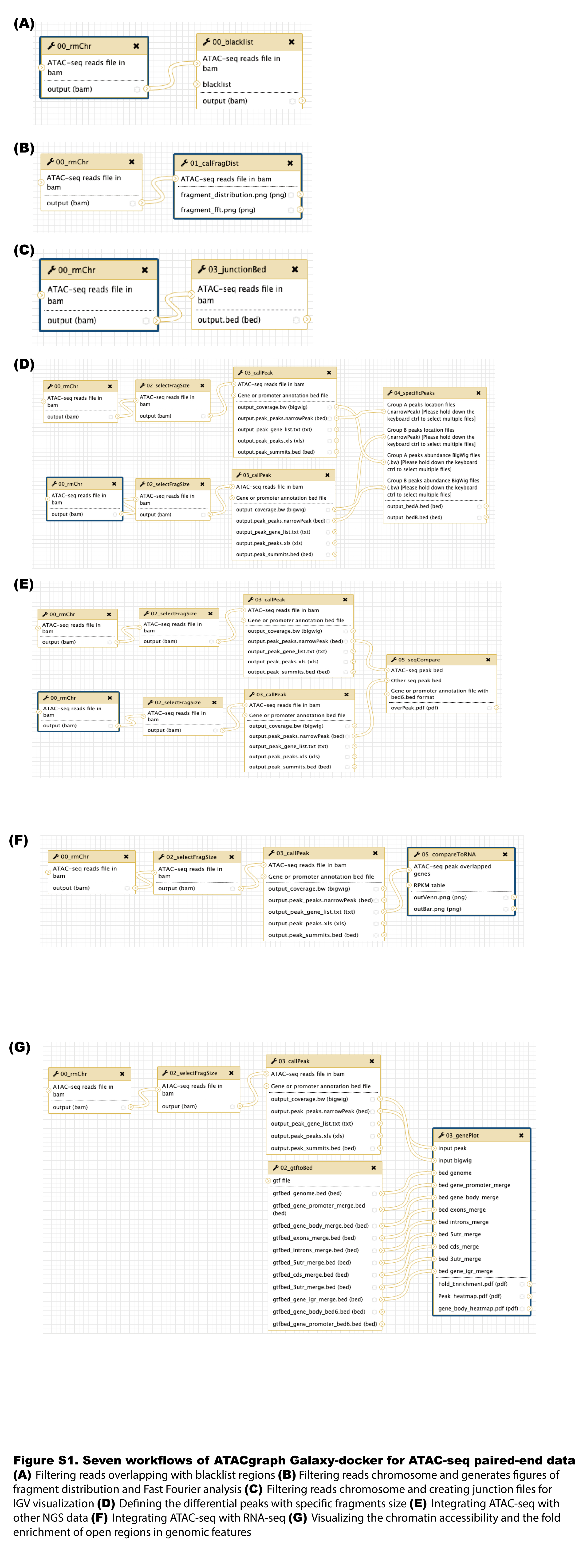

Supplement: Supplementary Figure 1 — Seven workflows of ATACgraph Galaxy-docker for ATAC-seq paired-end data analysis, (A) Filtering reads overlapping with blacklist regions. (B) Filtering reads chromosome and generates figures of fragment distribution and Fast Fourier analysis. (C) Filtering reads chromosome and creating junction files for IGV visualization. (D) Defining the differential peaks with specific fragments size. (E) Integrating ATAC-seq with other NGS data. (F) Integrating ATAC-seq with RNA-seq. (G) Visualizing the chromatin accessibility and the fold enrichment of open regions in genomic features. [file Image_1.jpg]
