# Supplementary material for: ATACgraph: Profiling Genome-Wide Chromatin Accessibility From ATAC-seq
Source: Front Genet. 2021 Jan 13;11:618478. doi: 10.3389/fgene.2020.618478 (PMC7874078; doi:10.3389/fgene.2020.618478)
Supplement: Supplementary Table 1 — Functions implemented in the ATACgraph. [file Table_1.PDF]

Table S1 Functions implemented in the ATACgraph

	Module Name	Usage Descriptions
**Preprocessing**	00_rmChr	Removing mitochondria/plastid DNA reads
	01_calFragDist	Plotting size distribution and calculating periodicity of sequenced fragments in ATAC-seq
	02_gtftoBed	Converting a GTF file to 8 BED files
	02_selectFragSize	Selecting reads with fragment length for down-stream analysis
**Profiling**	03_callPeak	Peak calling
	03_genePlot	Generating graphical summary results of chromatin accessibilities
	03_junctionBed	Generating an IGV track to display nucleosome free regions
**Comparison**	04_specificPeaksIDR	Identifying differentially accessible peaks between two groups
	04_specificPeaks	Identifying differentially accessible peaks between two groups
	04_specificPromoter	Identifying differentially accessible promoters between two groups
	05_seqCompare	Comparing ATAC-seq peaks with another type of NGS data
	05_compareToRNA	Comparison of accessible genes and genes expression
