# Supplementary material for: ATACgraph: Profiling Genome-Wide Chromatin Accessibility From ATAC-seq
Source: Front Genet. 2021 Jan 13;11:618478. doi: 10.3389/fgene.2020.618478 (PMC7874078; doi:10.3389/fgene.2020.618478)
Supplement: Supplementary Table 2 — Summary of suggested parameters for fragment length selection and peak calling. [file Table_2.PDF]

Table S2. **Summary of suggested parameters for fragment length selection and peak calling**

Identified region	Parameter selection for 02_selectFragSize module	Parameter selection for 03_callPeak module
Accessible regions	-	integration site
Nucleosome free region (NFR)	short fragment	full-extended fragment
Nucleosome occupied region	long fragment	full-extended fragment
