# Supplementary material for: ATACgraph: Profiling Genome-Wide Chromatin Accessibility From ATAC-seq
Source: Front Genet. 2021 Jan 13;11:618478. doi: 10.3389/fgene.2020.618478 (PMC7874078; doi:10.3389/fgene.2020.618478)
Supplement: Supplementary Table 3 — Command list for mapping steps. [file Table_3.PDF]

Table S3 command list for mapping steps

Function	Command line
Bowtie2 mapping	*bowtie2 -p 30 -x genome_ref --no-unal --non-deterministic --no-mixed --no-discordant --very-sensitive --maxins 2000 -1 sample_R1.fastq.gz -2 sample_R2.fastq.gz > sample.sam*
SAMtools remove low quality reads	*samtools view -Sb -q 10 sample.sam sample.bam*
SAMtools sorting	*samtools sort sample.bam sample_sort.bam*
SAMtools remove duplicates reads	*samtools rmdup sample_sort.bam sample_uniq.bam*
